# Risk of Human Papillomavirus Infection in Cancer-Prone Individuals: What We Know

**DOI:** 10.3390/v10010047

**Published:** 2018-01-20

**Authors:** Ruby Khoury, Sharon Sauter, Melinda Butsch Kovacic, Adam S. Nelson, Kasiani C. Myers, Parinda A. Mehta, Stella M. Davies, Susanne I. Wells

**Affiliations:** 1 Divisions of Bone Marrow Transplantation and Immune Deficiency, Cincinnati Children’s Hospital Medical Center, Cincinnati, OH 45229, USA; Ruby.khoury@cchmc.org (R.K.); adam.nelson@cchmc.org (A.S.N.); kasiani.myers@cchmc.org (K.C.M.); parinda.mehta@cchmc.org (P.A.M.); stella.davies@cchmc.org (S.M.D.); 2 Divisions of Asthma Research, Cincinnati Children’s Hospital Medical Center, Cincinnati, OH 45229, USA; sharon.sauter@cchmc.org (S.S.); Melinda.Butsch.Kovacic@cchmc.org (M.B.K.); 3 Divisions of Oncology, Cincinnati Children’s Hospital Medical Center, Cincinnati, OH 45229, USA

**Keywords:** human papillomavirus, Fanconi anemia, orphan disease, squamous cell carcinoma, inherited cancer susceptibility

## Abstract

Human papillomavirus (HPV) infections cause a significant proportion of cancers worldwide, predominantly squamous cell carcinomas (SCC) of the mucosas and skin. High-risk HPV types are associated with SCCs of the anogenital and oropharyngeal tract. HPV oncogene activities and the biology of SCCs have been intensely studied in laboratory models and humans. What remains largely unknown are host tissue and immune-related factors that determine an individual’s susceptibility to infection and/or carcinogenesis. Such susceptibility factors could serve to identify those at greatest risk and spark individually tailored HPV and SCC prevention efforts. Fanconi anemia (FA) is an inherited DNA repair disorder that is in part characterized by extreme susceptibility to SCCs. An increased prevalence of HPV has been reported in affected individuals, and molecular and functional connections between FA, SCC, and HPV were established in laboratory models. However, the presence of HPV in some human FA tumors is controversial, and the extent of the etiological connections remains to be established. Herein, we discuss cellular, immunological, and phenotypic features of FA, placed into the context of HPV pathogenesis. The goal is to highlight this orphan disease as a unique model system to uncover host genetic and molecular HPV features, as well as SCC susceptibility factors.

## 1. HPV Infection, a Significant Threat to Public Health

Human Papillomaviruses (HPVs) are a group of more than 150 related viruses which are known to cause 5% of all human cancers by infecting keratinocytes in the skin and mucosas [1,2,3]. Cutaneous HPV types are widely present in the normal human skin and may contribute to skin cancer in cooperation with UV radiation [4]. Mucosal HPV types are the most common sexually transmitted viruses in the US [5]. There are two major HPV subtypes: high-risk types (e.g., HPV16 and 18) which cause anogenital and oropharyngeal squamous cell carcinomas (SCCs), and low-risk types (eg, HPV6 and 11) which cause genital warts and recurrent respiratory papilloma. Although most individuals infected with HPV clear the virus, a minority harbor persistent high-risk HPV which puts them at risk of HPV-associated diseases, including cancer. HPV-related health-care costs are exorbitant and, despite three Food and Drug Administration (FDA)-approved HPV vaccines, are likely to remain high for decades for a number of reasons: (a) individuals who are already infected are likely not protected by the vaccine for that particular subtype, (b) vaccine coverage is poor—in part due to racial, ethnic, and income disparities, and (c) a long latency separates infection from tumorigenesis [1,2,6,7,8]. Furthermore, effective cures for the infection are not available. While HPV-specific antivirals are greatly needed, their design has been hindered by a paucity of information about the viral life cycle—the molecular interactions between HPV and the heterogeneous keratinocytes that permit infection and induce and sustain virus amplification in the human epidermis [9,10,11,12].

The human epidermis is composed of four distinct layers. The deepest, basal layer contains epidermal stem and progenitor cells (ESPCs), keratinocytes whose proliferative activity is tightly regulated to regenerate the cells that are shed from the surface. Keratinocyte connectivity to the basement membrane and the underlying dermis occurs via hemidesmosomes. Despite that connectivity, the ESPC progeny exit the basal layer continuously and migrate outward through the suprabasal spinous, granular, and cornified layers, where they reside as terminally differentiated keratinocytes. Granular and cornified keratinocytes form an impermeable barrier to resist physical, chemical, and infectious insults. For example, the disruption of tight junctions by two Human Immunodeficiency Virus (HIV) proteins promotes HPV pseudovirus penetration, thus linking cellular adhesion defects to HPV infection [13]. Overall cell–cell connectivity is crucial for adherence and communication in the epidermis and is provided by intercellular junctions: desmosomes, adherence, gap, and tight junctions. Desmosomes are abundant throughout the epidermis and anchor to the intermediate filament cytoskeleton to ensure proper architecture and mechanical resistance. Desmosomes also function as signaling centers, regulating fundamental processes such as proliferation, migration, and morphogenesis [14,15,16]. In the cornified layer, desmosomes mature into corneodesmosomes, key components of the epidermal barrier function together with the tight junctions in the granular layer [17].

HPV virions contain circular double-stranded DNA genomes of approximately 8 kbp within an icosahedral capsid. For many high risk HPV types, the viral genome encodes six early (E) nonstructural, and two late capsid proteins (L1, L2). HPV gene expression is complex, involving a synchronization of transcription, mRNA stability, splicing, and polyadenylation with keratinocyte differentiation and distinct phases of the viral life cycle [18,19]. In the initial phase of the HPV life cycle, basal keratinocytes are infected by the virus that has permeated the above epidermal barrier through microwounds. The second phase is characterized by a short burst of viral genome amplification, followed by maintenance at 50–200 copies per cell. Upon upward migration and differentiation, the third phase, i.e., genome amplification, produces thousands of HPV copies in a fraction of keratinocytes in the granular and cornified layers. L1 and L2 expression and virus assembly are limited to these terminally differentiated layers. The switch from phase 2 (viral maintenance) to phase 3 (productive replication) is poorly understood, but is key to HPV disease and transmission, as it leads to the production of viral progeny that will infect the same or a different host. Therefore, the identification of regulators of HPV infection and amplification is a critical step towards new approaches to attenuate viral production. This review highlights how a DNA repair pathway, the Fanconi anemia pathway, interacts with HPV at a molecular, cellular, and epidemiological level and can help further our understanding of HPV pathogenesis and host response to identify potential targets for novel therapeutic interventions. As most of the studies cited below tested high-risk HPV subtypes, from this point on “HPV” will be used to refer to high-risk HPV, unless otherwise indicated.

## 2. Fanconi Anemia, an Inherited DNA Repair Syndrome

**Figure 1:**
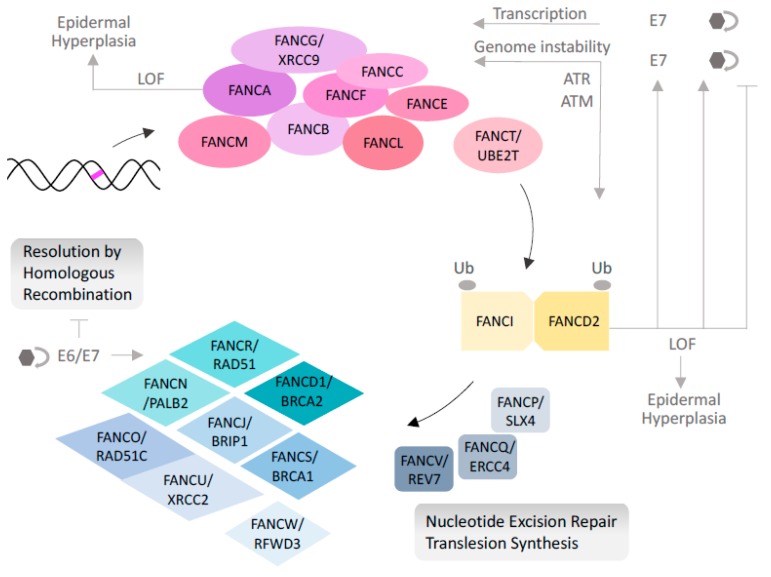
Only the classical role of the Fanconi anemia (FA) pathway in the repair of interstrand cross-links (ICLs) is depicted, which involves highly coordinated protein–DNA and protein–protein interactions in the nucleus. Other reported functions of the FA pathway in replication stabilization, origin firing, and cytokinesis are not shown, but are reviewed in detail in [22]. In response to ICL formation, the FA core complex (pink circles), including Fanconi Anemia Complementation Group (FANC) A (FANCA), assembles near the lesion, and this activation drives the ubiquitination, by FANCL, of two central pathway components FANCD2 and FANCI (yellow squares), and their recruitment. These initial steps require the activity of the ataxia-telangectasia mutated (ATM) and ataxia-telangectasia and Rad-3 related (ATR) DNA damage sensor kinases. The formation of the central "ID" complex is followed by the activity of endonucleases and translesion polymerases (grey squares) for ICL unhooking and bypass. Finally, the recruitment of homologous recombination proteins (blue diamonds) completes the repair process. Some examples of HPV interactions with the FA pathway are depicted in grey. Pink bar: interstrand cross link in the DNA double helix. Arrow: activation, hatched line: inhibition, hexagon: HPV, hexagon with circular arrow: HPV replication. LOF: loss of function. BRCA: Breast cancer susceptibility protein; PALB: Partner and localizer of BRCA2; BRIP: BRCA interacting protein; RFWD: ring finger and WD repeat domain protein; XRCC: X-ray repair cross complimenting protein; UBE2T: Ubiquitin conjugating enzyme E2 T; REV7: DNA polymerase zeta processivity subunit also known as MAD2L2 (mitotic arrest deficient 2 like 2); SLX: structure specific endonuclease subunit; ERCC: excision repair 1 protein.

Fanconi anemia (FA) is an inherited autosomal recessive, rarely X-linked or dominant negative, disorder characterized by congenital abnormalities, progressive bone marrow failure, and a predisposition to malignancies. FA arises from a germline loss of function of any one of 22 known FA genes involved in DNA repair. This pathway specializes in the repair of interstrand cross-links (ICL) (Figure 1), but is also implicated in stabilizing stalled replication forks, ensuring proper cytokinesis, and suppressing nonhomologous end-joining [20,21,22,23,24]. ICLs occur endogenously during the S phase or upon exposure to exogenous crosslinkers, such as chemotherapeutics, aldehydes, or other breakdown products from alcohol, tobacco, and dietary fats [25]. ICLs block transcription and replication near the lesion, and therefore their continuous removal is required for sustained cellular survival. In response to ICL formation, the protein products of eight FA genes assemble to form the nuclear “FA core complex” at the lesion, trigger the monoubiquitination of Fanconi Anemia Complementation Group (FANC) D2 (FANCD2) and FANCI, and then activate the FANCD2/FANCI dimer that orchestrates the coordinated recruitment of downstream proteins to repair the lesion. These include nucleolytic processing proteins, translesion polymerases, and homologous recombination proteins [26]. Two DNA damage sensor kinases, ataxia-telangectasia mutated (ATM) and Rad-3 related (ATR), play intricate roles in this process by phosphorylating and activating a number of FA proteins. Once the repair is completed, the deubiquitination of the FANCD2/FANCI complex by ubiquitin specific peptidase 1 USP1 and USP1- associated factor (USP1-UAF1) leads to complex release from chromatin and to pathway reset. Loss of the FA pathway results in the accumulation of ICLs and in global genome instability. The intact HPV genome is an episome [27]. However, HPV integration into the host genome and sustained expression of the two oncogenes E6 and E7 can also lead to genome instability, but additionally inactivate members of the p53 and Retinoblastoma (RB) tumor suppressor families, playing an important role in inducing carcinogenesis [28,29,30]. Genomic instability and accumulation of mutations in the context of the FA DNA-repair pathway dysfunction are likely contributors to FA patients’ susceptibility to HPV-independent and HPV-associated SCCs. Below, we summarize the clinical features of FA, the epidemiology of HPV in FA, and cellular mechanisms underlying altered HPV biology and perhaps HPV-associated SCC in FA.

## 3. The Many Phenotypes of Fanconi Anemia

FA is characterized by congenital abnormalities, progressive bone marrow failure, and a predisposition to leukemia and solid tumors. Congenital abnormalities occur in approximately 75% of patients with FA and commonly include short stature, abnormal skin pigmentation, skeletal malformations, and genitourinary tract abnormalities [31]. The age of the onset of bone marrow failure varies significantly, with a median age of 7.6 years [32]. Individuals with FA carry a higher risk of developing myelodysplastic syndrome (MDS), acute myelogenous leukemia (AML), or both. The risk of developing AML is increased 500-fold as compared to the general population, and most individuals are typically diagnosed between the ages of 15 and 35 [33]. The relative risk of developing MDS is even higher, 5000-fold greater than that of the general population [34]. The only curative therapeutic option for the hematologic manifestations of FA is a hematopoietic stem cell transplant (HSCT), which should ideally be performed prior to the onset of MDS and AML. HSCT outcomes have dramatically improved over the last 20 years because of the attenuation of the preparative regimen intensity overall, with the addition of fludarabine and the elimination of total body irradiation. The five-year survival after a matched sibling transplant is now close to 90%, and excellent outcomes have also been achieved with alternate donor transplants, especially in patients under the age of 10 years [35,36,37].

While a successful stem cell transplant can cure the hematologic manifestations of FA, including bone marrow failure, AML, and MDS, post-HSCT individuals remain at a significantly increased risk for solid tumors. The most common tumors seen in FA are head and neck squamous cell carcinomas (HNSCC), particularly in the oral cavity, and vaginal squamous cell carcinomas, which occur at a younger age and are very challenging to treat because of the inherent chemotherapy and radiation therapy sensitivity of individuals with FA. A cohort study of 35 individuals with FA and HNSCC from the International Fanconi Anemia Registry reported a low overall 5-year survival of 39%, with 49% of individuals with HNSCC experiencing recurrence after treatment [38]. In this study, 15 out of 20 tumors that were tested for HPV were positive (75%) [39]. One hypothesis for the predisposition of individuals with FA to SCCs in the oral cavity and genital region is an increased susceptibility to exogenous carcinogens, including oncogenic viruses such as HPV. A subpopulation of tumors harbor HPV sequences, but the role of HPV in the development of SCC in patients with FA has been controversial [40]. Some studies have failed to detect HPV in HNSCCs derived from patients with FA, others have found a high prevalence of HPV positivity in both FA-related HNSCC tumors and oral samples from individuals with FA [39,41,42,43,44]. While the evidence implicating HPV in HNSCC is controversial, there is a clearer association with a subset of anogenital SCCs. More studies are needed to establish whether HPV plays a role in the development of many or most SCC in patients with FA, and to determine whether FA imparts a greater likelihood of viral infection, replication and persistence, as might be indicated by experimental data.

## 4. FA Loss Stimulates HPV Genome Amplification, Integration, and Oncogenicity

Early studies of HNSCC, anogenital warts, and other SCCs in individuals with FA suggested a possible role for HPV in FA-associated cancers [39]. This report was consistent with another US-based study that found HPV DNA prevalence to be significantly greater in 25 FA-related HNSCC tumors compared to non-FA controls [41]. However, a Dutch study failed to detect HPV DNA in any of the FA-related tumors tested, and HPV was not detected in five HNSCC or four anogenital squamous cell carcinoma samples from subjects with FA in a more recent report [41,42]. While the detection of HPV in FA HNSCC remains controversial, there is growing evidence from cell lines, animal studies, expression analysis, and computational biology approaches that supports a virus–cellular crosstalk between the FA pathway and HPV infection and oncogenicity [45]. HPV interacts with a number of DNA damage pathways. For instance, the E1 and E2 replication proteins activate a DNA damage response which includes ATM and ATR signaling. The E6 and E7 oncogenes promote viral replication by both activating and deactivating DNA damage repair pathway components and by uncoupling inappropriate cell cycle progression from apoptosis. Detailed HPV interactions with DNA damage repair pathways are described in [46]. Our focus below remains on the FA pathway.

First, microarray analysis of vulvar tissue infected with both noncarcinogenic and carcinogenic HPV types indicated that FANCA, FANCD2, and other DNA damage markers were significantly induced following high-risk HPV infection, along with increased DNA damage in the tissues [47]. Similarly, the expression of the high-risk HPV16 E7 oncogene, individually or together with E6, led to a coordinated upregulation of FA genes by E7 expression via Rb/E2F (retinoblastoma/E2F transcription factor 1) regulation [48]. Second, a link between HPV16 and the FA DNA repair pathway was identified using a computational biology approach and then validated experimentally by evaluating the impact of overexpressing the E6 or E7 proteins in primary fibroblasts and keratinocytes, using global gene expression analysis [45]. Third, the FA pathway loss in human HPV16+ or HPV31+ organotypic epithelial rafts increased DNA damage as expected from the loss of a key DNA repair pathway. However, quite unexpectedly, cell proliferation, expansion of the basal ESPC compartment, and tissue hyperplasia were observed. FA correction rescued the abnormal phenotype [49,50]. Increased cellular and viral replication were correlated with elevated levels of the E7 oncoprotein, but relevant host–viral networks whereby the FA pathway limits HPV genome amplification and thus, perhaps, infectious virus yields are unclear [46]. In subsequent experiments with these models, FANCD2 loss stimulated HPV16 and HPV31 genome amplification on the basis of qPCR and in situ hybridization—including in the basal and spinous cell layers where genome amplification does not normally occur. It is likely that the stabilization of the viral E7 protein contributes to both viral and cellular hyper-replication [49]. Since increased viral and cellular proliferation in 3D epidermis was an immediate response to FA loss, these phenotypes are not likely a consequence of adaptation by selection. Fourth, in the context of an intact FA pathway, the central FANCD2 protein was recently shown to localize to HPV viral replication centers, and FANCD2 knockdown promoted integration at the expense of viral replication [51]. Thus, depending upon the experimental circumstances, FA loss can lead to either amplified viral replication or increased integration with reduced replication. Regardless of the outcome of these processes, increased viral load or integration, would be expected to stimulate neoplastic transformation. Fifth, the HPV E6 and E7 oncoproteins repress homologous replication [52]. Sixth, *Fancd2* knockout mice do not develop SCC spontaneously and, therefore, are not a model for human SCC susceptibility in the absence of other gene modifications or environmental carcinogens. However, *Fancd2* knockout mice, bred to mice with transgenic expression of the HPV16 E7 oncoprotein targeted to basal epithelial cells, harbor increased DNA damage in mutagen-treated epidermis and are more likely to develop head and neck SCCs [53], cervical, and vaginal SCCs, compared to E7-transgenic control animals [54]. These effects of E7 are due to the inactivation of the Rb family of tumor suppressors that normally limit DNA damage [55]. Altogether, a multitude of physical, molecular, and functional connections between the FA pathway and HPV oncogenes in epidermal models may support a clinically important relationship in humans.

Together, these diverse data point to a common theme. It appears that HPV infection results in elevated DNA damage that then triggers the FA pathway to repair this DNA damage [56] and reprograms the FA pathway to participate in viral genome processing. In individuals where this pathway is defective, it is likely that the DNA damage will not be repaired in HPV E7-expressing, highly proliferative cells, compounding the likelihood of tumor development over time. For these reasons, it is now critical to reconsider these studies in the context of the conflicting human data. Even if HPV is suppressed or cleared to levels undetectable by PCR assays, one might speculate that the resulting DNA damage is the trigger for increased HNSCCs and anogenital carcinomas clinically evident years later. This may have relevance for sporadic tumors where the FA pathway is frequently inactivated, either mutationally or through transcriptional silencing. Exome sequencing data and whole genome sequencing data demonstrated that 11% and 18%, respectively, of both HPV+ and HPV- HNSCCs in the general population harbor nonsynonymous mutations in FA genes [57,58], suggesting selective pressure for FA pathway loss during tumor development or progression. The depletion of components of the FA pathway in sporadic HPV-positive and -negative HNSCC cell lines induced epithelial to mesenchymal transition (EMT)-like phenotypes and invasion, features of advanced tumors, by mechanisms that involve the activation of the DNA-PK (DNA-protein kinase) DNA damage sensor kinase and downstream signaling through the Rac1 GTPase (Rac Family Small GTPase1) [58]. Collectively, there is impressive evidence pointing to a role for HPV in FA SCC, and a role for HPV-independent phenotypes, including DNA damage induction and cellular tumor phenotypes. Despite this, etiological associations remain unproven, and studies of the natural history of tumor development in the HPV-positive (and -negative) hosts are now needed to identify the underlying mechanisms of infection by HPV and to explore the role of other viruses or pathogens as possible contributors to cancer risk. Intriguingly, recent in vivo data from the Lambert laboratory may even hint at a possible hit-and-run mechanism for SCC development following high-risk HPV infection. This 2016 study again used HPV16 E7 transgenic mice, wherein the transgene expression is conditionally controlled [59]. Following the conventional paradigm in *Fancd2*-proficient mice, the persistence of cervical neoplasia was highly dependent upon the continued expression of HPV16 E7. In *Fancd2*-knockout mice, however, cervical cancers persisted after HPV16 E7 expression was turned off, suggesting that FA loss relieves tumors from sustained E7 dependency. Since HPV oncogenes cause an accumulation of DNA damage, the authors hypothesized that HPV-induced DNA damage leads to an accumulation of mutations in FA-deficient mice, which might allow HPV-driven cancers to acquire a rapid independence from the viral oncogenes. If true for individuals with FA, scenarios of hit-and-run HPV infection would arise for situations where the FA pathway is inactivated. This paradigm would be consistent with the ongoing debate about HPV detection in SCC specimens from the FA population and would explain past difficulties to establish or rule out HPV causality in FA SCC. Immune dysfunction in patients with FA may also play a role in cancer development in addition to the underlying genome instability and the inability of the cells to repair the DNA damage caused by HPV16 infection.

## 5. Immune dysfunction in FA and HPV susceptibility

To date, only limited studies have been published investigating the immune function in children and adults with FA. Froom et al. reported a low natural killer (NK) cell activity in two FA patients and their family members, suggesting a potential intrinsic defect in NK cell activity associated with FA [60]. Three additional studies subsequently supported these findings [61,62,63]. High levels of tumor necrosis factor alpha (TNF-α) [64,65] and low production of interleukins (IL), including IL1, IL2, and IL6, as well as interferon gamma (IFN-γ) and granulocyte-macrophage colony-stimulating factor (GM-CSF) have also been reported, while immunoglobulin (Ig) levels were shown to be within the normal range [66]. Another study evaluated 11 individuals with FA and found their peripheral blood mononuclear cells (PBMCs) to have lower lymphoproliferative responses to phytohemagglutinin (PHA) and pokeweed mitogens compared to controls, implying that they may have a general immune defect [67]. Further, this study showed that PBMCs of individuals with FA responded poorly compared to controls to a stimulation with Tetanus toxoid and a purified protein derivative of mycobacterium, but not cytomegalovirus (CMV), antigen, suggesting that individuals with FA may have lower activation and proliferation capabilities, and therefore increased susceptibility to some, but not all, infections. Using flow cytometry to study PBMCs, Castello et al., reported the potential to grade immunologic defects in individuals with FA, as well as in their asymptomatic parents (obligate heterozygotes), based on the differential expression of cell surface markers in lymphocytes, including cluster of differentiation (CD)20, CD4, CD8 (reflected in the CD4/CD8 ratio), CD25, and HLA-DR (Human leukocyte antigen- DR) [68]. Most compelling in this study was the activated status of T cells in patients and their parents, on the basis of CD25 and HLA-DR expression [60].

Similarly, Petridou et al. studied the parents of individuals with FA and found low levels of NK cell subsets as well as reduced mitogen-induced proliferation of PBMCs [62]. Another group observed a decrease in NK CD56dimCD16+ and CD8+ T cells in FA *(n* = 42) compared to non-FA controls [69]. These data further imply that the impaired differentiation of the NK cells subsets may be directly related to the impairment of the immune surveillance of viruses. Our own retrospective, cross-sectional analysis of a small group of children with FA (*n* = 10) showed a heterogeneous immune dysfunction compared to non-FA children [70]. Overall, we found that children with FA had decreased numbers of natural killer (NK) cells with impaired function (decreased NK lytic units and perforin and granzyme levels), fewer CD19+ B cells and tetanus responses, and diminished cytotoxic T lymphocyte (CTL) function [70]. A more recent study (*n* = 31) found that FA adults, but not children, had significantly lower IgG, IgA, IgM, total lymphocytes, and CD4 T cells than their relatives or compared to reference values (*p* < 0.001) [71]. Consistent with our study, this study found that both children and adults with FA had fewer B- and NK cells compared to controls.

More recently, we characterized the immune competence of a larger cohort of individuals with FA and assessed lymphocyte populations and functional status in the blood of 29 pre-BMT (bone marrow transplant) individuals with FA (2–47 years), with no history of cancer or severe bone marrow failure [72]. Strikingly, few individuals with FA were normal in all parameters tested. Many individuals with FA had reduced total NK cells, confirming the previously observed decreases in absolute CD16+ NK cells in FA. In those with normal NK levels, NK function was significantly impaired. Similar to previous results, decreased absolute CD19+ B cells were observed, but further investigation revealed also fewer CD19+CD27+ memory B cells and lower levels of immunoglobulins. In fifteen individuals with corresponding clinical bone marrows, we also compared blood immune parameters to bone marrow parameters. Not unexpectedly, we observed a strong positive correlation (*r* = 0.96) between mature B cell percentages in the bone marrow and CD19+ B cells in the blood, suggesting that the peripheral blood represents the bone marrow well. Further, we observed a moderate negative correlation (*r* = −0.54) between mature B cell % in the bone marrow and CD19+CD27+ memory B cells in the blood, suggesting that memory B cells may remain in the bone marrow longer, unlike what is often observed in individuals with typical immune deficiencies. While total CD3+ T cells and CD8+ T cells were not significantly different, we observed fewer CD4+T cells and reduced CTL function in individuals with FA. Since other laboratories had observed aberrant CD8+ T cells in FA, we took a closer look at the regression of the CD8+ *z*-scores by time and stratified by age. Indeed, the subanalysis uncovered more CD8+ T cells in older FA subjects (*p* = 0.0002), who also had fewer CTL lytic units (*p* = 0.03).

Taken together, these findings point to potential alterations of the immune function in individuals with FA and in their parents and provide support for an immunologic basis of the viral susceptibility, or susceptibility to other pathogens observed in FA. Importantly, a reduced NK cell activity may critically alter immune surveillance with regard to neoplastic cells in a population with known predisposition to DNA damage and malignancy. The differences in the study populations (median cohort age, status of bone marrow function, percentage having had a BMT, etc.) likely contribute to the variable findings between studies. Longitudinal studies are needed to determine whether the observed immune defects are present at birth or develop with age.

## 6. Epidemiological Studies in Fanconi Anemia Demonstrate Increased Oral HPV Prevalence

Currently there are no known published longitudinal studies of HPV in people living with Fanconi anemia. However, epidemiological studies have provided some evidence of risk. One study collected oral swabs from 67 participants with Fanconi anemia and tested for 27 HPV genotypes using polymerase chain reaction-based methods [73]. The study reported that the prevalence of oral HPV infection was 7.5%, and the prevalence of high-risk HPV infection was 6.0%. The prevalence was higher in adult males than in adult females (25.0% versus 9.1%, respectively). Another related study collected oral rinse samples from 126 individuals with FA and 162 unaffected first-degree relatives, testing for 37 HPV types [44]. The study found that 11.1% of individuals with FA tested HPV+, a percentage that was significantly higher (*p* = 0.003) than that corresponding to the primary relatives (2.5%). The HPV prevalence was even higher for sexually active individuals with FA (17.7% versus 2.4% for the family members; *p* = 0.003). HPV positivity also tended to be higher in the sexually inactive (8.7% for FA versus 2.9% for their siblings). Indeed, having FA increased HPV positivity 4.9-fold (95% confidence interval (CI): 1.6–15.4), considering age and sexual experience, but did not affect other potential risk factors. Among the 14 individuals with Fanconi anemia who tested positive for oral HPV, 8 individuals had corresponding immune data [44]. Only three of the eight oral HPV+ subjects had been vaccinated for HPV prior to blood sampling and all had positive titers of all four HPV types included in the Gardasil vaccine. Two of the oral HPV+ individuals who indicated that they had not yet been vaccinated for HPV were also serologically HPV+ for types other than those identified in their oral sample, suggesting prior natural infections with these types. Three of the oral HPV+ participants had no detectable HPV titers. Two individuals were HPV6+ and HPV16+. Interestingly, these individuals were deficient in either their absolute B cell count or their memory B cells count. While there were no unifying characteristics that were shared by oral HPV or seropositive individuals with FA, these data support that individuals with FA have heterogeneous and frequently reduced immune responses [70,72]. Considering these oral results, even young children with FA are commonly infected with HPV, likely from nonsexual routes. A recent study found that HPV can survive outside of its host to potentially infect people by nonsexual means [74]. Testing for HPV in multiple anatomical sites at all ages and particularly before and after vaccination could inform vaccine guidelines for FA and for other cancer-prone and immunodeficient populations. A continued study of both immunity and HPV infection is needed to better understand their contributions to SCC.

## 7. Response to HPV Vaccine in Individuals with FA

Three HPV vaccines are currently licensed: two vaccines containing L1 Virus-Like Particles (VLPs) for HPV types 6, 11, 16, and 18 (Gardasil and Gardasil-9, which additionally covers HPV types 31, 33, 45, 52, and 58 (Merck and Co., USA), and a third vaccine containing L1 VLPs for HPV types 16 and 18 (Cervarix, GlaxoSmithKline Biologicals, Belgium, which has since been withdrawn by GSK from the US market) [75]. The impaired B cell function in FA raises concern about the efficacy of vaccination in FA. Our earlier study of the immune function in FA tested the type-specific seropositivity using a bead Luminex assay. Seropositivity ranged from 7 to 21% for skin HPV types and from 7 to 38% for mucosal HPV types in unvaccinated individuals with FA; in self-reported vaccine recipients, the serological positivity to HPV vaccine types was 75 to 96% [76]. Our more recently published study of the immune response in FA reported antibody responses to natural HPV infection and HPV vaccination in 39 unvaccinated and 24 vaccinated patients for HPV [77]. Similar to the earlier study, 30% of reportedly unvaccinated individuals were seropositive. As expected, seropositivity was significantly associated with having had sex regardless of age (*p* = 0.03). Importantly, among the 23 unvaccinated children younger than 13 years old, 5 were positive to more than one HPV types by multiplex ELISA (M4ELISA—HPV16, 18, 6, 11). Seropositivity among individuals vaccinated for HPV16, 18, 6, and 11 was also high. However, HPV titers for all four subtypes were significantly lower in the post-HSCT participants compared to those who had also received the vaccine but had not undergone HSCT. Currently, there are few other published US studies that have reported HPV titers for individuals with FA. One study of 34 unvaccinated and 12 mainly Gardasil vaccinated individuals with FA suggests that individuals with FA are, to some level, protected by the vaccines up to 5 years, similar to the general population [78]. While none of the immune function measures predicted whether the participants’ responses would be in the expected range for their age or whether they would have lower HPV titers following vaccination, 23% of participants (*n* = 3) with lower HPV titers had a low total memory B cell count, while none of the participants with titers in the expected range had a low memory B cell count. Indeed, this group tended to have more participants with a higher memory B cell count, suggesting a possible association between preserved memory B cells and HPV titer. Further study of host immune responses is needed to determine whether inherent virus-specific immune defects contribute to persistent HPV infection.

## 8. Epidermal abnormalities in Fanconi Anemia

A prominent feature of FA is an abnormal pigmentation of the skin and mucosas, and, as mentioned previously, individuals harbor an extreme disposition to keratinocyte-based SCCs early in life, primarily oral, esophageal, and anogenital tumors, as well as skin tumors [39,79,80,81,82]. First, individuals with FA have a significantly increased risk of HPV positivity (compared to unaffected family members) [44]. Second, the viral presence or the seropositivity for cutaneous and mucosal HPV types is detectable in FA individuals [43,73,76]. Anogenital SCCs in FA are linked to a history of low-risk HPV infection-associated genital warts; a causal role for the high-risk HPV types 16 and 18 in FA head and neck SCCs remains controversial [38,39,41,42,43,73,76,78,83,84,85,86,87]. Several laboratories have published clinical data that suggest that FA genes limit mucosal and cutaneous HPV infection and amplification. It is unclear whether frequent pigmentation defects are associated with any subclinical skin vulnerabilities.

Interestingly, an unrelated SCC predisposition syndrome, epidermolysis bullosa (EB), is known for defective collagen production, or defects in cell–cell or cell–substratum adhesion complexes which result in skin fragility, skin blistering and scarring, and increased risk of SCC formation [88,89]. Our published and preliminary data suggest the presence of adhesion defects in SCCs and perhaps the skin of individuals with FA [90]. However, unlike individuals with EB, those with FA do not exhibit any clinically evident skin blistering at baseline. Defects in the structure or barrier function of the skin may allow exogenous stressor permeation into the deeper layers of the skin, further stimulating the existing genetic instability, progression to dysplasia, and cancer development. Similarly, in these individuals, the skin is a target organ for viral infection (including but not limited to HPV), as seen in other skin fragility conditions with increased SCC development, where reduced T cell numbers allow HPV to overcome host defenses and contribute to malignancy [91,92]. In the FA-deficient HPV immortalized epidermis, an increased epithelial hyperplasia was observed suggesting a role for the FA pathway in the maintenance of alternative epithelial properties, such as its structure and cell cycle control for the prevention of HPV-associated SCCs [50]. There may be other nonstructural abnormalities of the epithelium that predispose to the development of SCCs in individuals with FA. Naturally occurring aldehydes are known to damage the DNA by ICLs, and relevant repair mechanisms are deficient in individuals with FA. Acetaldehyde-mediated carcinogenesis has been reported in the squamous epithelium, and aldehyde dehydrogenase-deficient keratinocytes demonstrate increased aldehyde-derived DNA damage [93,94].

Another important question is whether keratinocyte abnormalities in individuals with FA contribute to growth factor abnormalities that could be involved in the growth of tumors or in SCC development. Individuals with FA commonly have pigmentation abnormalities of the skin, with café-au-lait or hypopigmented areas noted on clinical examination. Neurofibromatosis (NF) Type 1 (NF-1) is associated with café-au-lait lesions of the skin. Elevated levels of hepatocyte growth factor and stem cell factor were demonstrated in cultured fibroblasts of individuals with NF compared to controls and in keratinocytes from non-café-au-lait lesions in individuals with neurofibromatosis [95]. Keratinocytes from café-au-lait lesions in non-FA individuals were shown to produce elevated levels of endothelin-1 [96]. Deregulated levels of expression of potential growth-related cytokines may promote tumor development in individuals with the inability to repair DNA damage. However, further studies on the secretion of growth factors from FA-keratinocytes are needed, as are expanded epidemiological studies on the potential skin SCC susceptibility in FA.

Taken together, there may be an integral role for epidermal structural and functional abnormalities in the development of SCCs in individuals with FA. If true, this could significantly compound the effects of inherent genome instability by increasing the risk of mechanical stress or of carcinogen exposure. A multifactorial network involving potentially increased skin fragility, antigen entry, growth factor secretion, and the inability to effectively repair DNA damage may coalesce to foster SCC development in these individuals. Further work exploring the mechanisms behind these skin defects and the possibilities of therapeutic prevention or intervention is crucial to prevent the life-limiting complication of SCC in FA.

## 9. Summary

Individuals with Fanconi anemia have a dramatically increased risk of developing SCC when compared to the general population, especially as more FA individuals are surviving into adulthood because of improved outcomes due to HSCT. High-risk HPVs have been found to be causative agents of SCCs in the general population, with subtype 16 alone found to be present in 90% of HPV-related HNSCCSs. The association between HPV and SCCs in individuals with FA remains controversial. While there has been conflicting clinical evidence linking HPV positivity to HNSCCs, HPV seems to be more common in anogenital SCCs in this patient population.

While there are a few studies with a limited sample size assessing the immune function in individuals with FA, these studies have shown that individuals with FA do have some defects in cellular and humoral immunities. There are also studies showing that the FA pathway limits cellular proliferation and the amplification and integration of HPV. Taken together, multiple factors might predispose individuals with FA to HPV infection and oncogenicity. This might not have been reliably reproduced in the literature for various reasons. One reason is that, according to studies from the Kovacic laboratory, there are more types of HPV that might be implicated in HNSCC but that were not tested in human tumors. Another reason is that, perhaps, HPV infection is less implicated in the development of SCCs, particularly in the head and neck, in individuals with FA as compared to the general population. In this case, other factors ranging from other oncogenic pathogens to DNA damaging exposures might be at play. Yet another hypothesis is that, perhaps, the initial HPV infection supports the development of SCCs by a hit-and-run mechanism, in the absence of HPV persistence. We believe that more epidemiological and basic research studies are needed to study the prevalence of HPV in individuals with FA and the possible predisposing factors, such as defects in immunity and the loss of proliferative control and epidermal integrity that might be at play.
